# Positron annihilation spectroscopy study of radiation-induced defects in W and Fe irradiated with neutrons with different spectra

**DOI:** 10.1038/s41598-020-75737-8

**Published:** 2020-11-03

**Authors:** O. V. Ogorodnikova, M. Majerle, J. Čížek, S. Simakov, V. V. Gann, P. Hruška, J. Kameník, J. Pospíšil, M. Štefánik, M. Vinš

**Affiliations:** 1 National Research Nuclear University “MEPHI” (Moscow Engineering Physics Institute), Kashirskoe sh. 31, Moscow, Russia; 2 Nuclear Physics Institute of the CAS, Řež 130, 250 68 Řež, Czech Republic; 3 Department of Low-Temperature Physics, Charles University, V Holešovičkách 2, 180 00 Prague, Czech Republic; 4 Institute for Neutron Physics and Reactor Technology, Karlsruhe Institute of Technology, Hermann-von-Helmholtz-Platz 1, 76344 Eggenstein-Leopoldshafen, Germany; 5 National Science Centre “Kharkov Institute of Physics and Technology”, Kharkov, Ukraine; 6 Department of Condensed Matter Physics, Charles University, Faculty of Mathematics and Physics, Ke Karlovu 5, 121 16 Prague 2, Czech Republic; 7 Research Centre Řež, Řež 130, 250 68 Řež, Czech Republic

**Keywords:** Nuclear fusion and fission, Metals and alloys, Experimental particle physics

## Abstract

The paper presents new knowledge on primary defect formation in tungsten (W) and iron (Fe) irradiated by fission and high-energy neutrons at near-room temperature. Using a well-established method of positron-annihilation lifetime-spectroscopy (PALS), it was found that irradiation of W in the fission reactor and by high-energy neutrons from the p(35 MeV)-Be generator leads to the formation of small radiation-induced vacancy clusters with comparable mean size. In the case of Fe, smaller mean size of primary radiation-induced vacancy clusters was measured after irradiation with fission neutrons compared to irradiation with high-energy neutrons from the p(35 MeV)-Be generator. It was found that one of the reasons of the formation of the larger size of the defects with lower density in Fe is lower flux in the case of irradiation with high-energy neutrons from the p(35 MeV)-Be source. The second reason is enhanced defect agglomeration and recombination within the energetic displacement cascade at high energy primary knock-on-atoms (PKAs). This is consistent with the concept of the athermal recombination corrected (arc-dpa) model, although the measured dpa cross-section of both fission neutrons and wide-spectrum high-energy neutrons in W is between the conventional Norgett–Robinson–Torrens (NRT-dpa) and arc-dpa predictions. This means that the physics of the primary radiation effects in materials is still not fully known and requires further study through a combination of modeling and experimental efforts. The present data serve as a basis for the development of an improved concept of the displacement process.

## Introduction

Tungsten (W) is considered as an attractive material for use in advanced fission and fusion reactors because of its high melting point, excellent high temperature strength, good thermal conductivity, low physical sputtering yield, relatively good resistance against radiation swelling, and good resistance against corrosion^1^. For these reasons, W is a leading candidate as a material that will be in direct contact with plasma in future fusion reactors, ITER and DEMO^1,2^. Reduced-activation ferritic-martensitic (RAFM) steels are considered as primary structural materials in advanced fission and fusion reactors^3–6^. To predict the behavior of materials under irradiation, it is important to understand primary defect formation.

The importance of controlling neutron-induced degradation of fission reactor materials has necessitated the study of radiation damage to metals over many decades. In future thermonuclear and advanced fission reactors, materials must withstand irradiation of high-energy neutrons. In the fusion reactor, materials will be irradiated with 14 MeV (in peak) neutrons generated by D–T fusion reaction. As a fusion neutron source does not exist yet, fission neutrons and charged particles are widely used to simulate fusion neutron-induced damage in materials, see, for example^7–9^**.** In early papers, the total density of radiation-induced defects in metals irradiated with electrons^10^, fission neutrons^11,12^ and fast neutrons from d(30 MeV)-Be source^13^ was measured using resistivity technique. However, data on the size distribution of radiation-induced defects were not reported. This is due to the fact that the resistivity experiments do not take into account agglomeration of primary radiation-induced defects into clusters of different sizes, although the size, shape and spatial distribution of defects can affect the material properties on a long-term time scale. Therefore, advanced microscopic studies are needed to understand what fraction of irradiated vacancies agglomerates into vacancy clusters, what is the morphology and size distribution of vacancy clusters, and what is the role of vacancy clusters in the deterioration of the macroscopic physical properties of irradiated materials.

A recent paper^14^ compared radiation-induced defects in W and iron (Fe) irradiated with high-energy protons and neutrons. Positron annihilation lifetime spectroscopy (PALS) was employed for characterization of size and concentration of radiation-induced defects^15^**.** PALS is an effective technique for detecting and characterizing radiation-induced defects that are often below the resolution limit of transmission electron microscopy (TEM). It was shown that 22.5 MeV protons produce mono-vacancies in W but the neutrons from the p(35 MeV)-Be generator produce vacancy clusters with an average size of 3–5 vacancies during irradiation at ambient temperature up to a fluence of 1.4 × 10^20^ n/m^2^ (corresponding to about 9 appm of the NRT-dpa). In the present paper, we extend the pioneer work^14^ by comparing the cluster size of vacancy-type defects formed in W and Fe after irradiation with fission and wide-spectrum high energy neutrons.

Experimental data on the total primary defect production under different types of irradiation can serve as a basis for comparison with models of the displacement process. The classical ‘NRT’ model developed in 1975 by Norgett, Robinson, and Torrens^16,17^ is used to estimate the displacement per atom (dpa) produced by different types of particles. The number of primary knock-on atoms *N*_*d*_ in the irradiated material can be calculated using the NRT-dpa model as follows:

1 $$N_{d} (T) = \left\{ {\begin{array}{*{20}l} {0,} \hfill & {{\text{if}}\quad T < E_{d} } \hfill \\ {1,} \hfill & {{\text{if}}\quad E_{d} < T < 2E_{d} /0.{\text{8}}} \hfill \\ {0.{\text{8}}T/({\text{2}}E_{d} ),} \hfill & {{\text{if}}\quad {\text{2}}E_{d} /0.{\text{8}} < T < \infty .} \hfill \\ \end{array} } \right.$$

In the above equation, *T* denotes the kinetic energy of reaction recoil which is transferred to the knocked atom by an elastic collision. The symbol *E*_*d*_ indicates the threshold of energy required to displace an atom from the lattice.

In general, the threshold displacement energy is a function of crystallographic orientation. However, the crystallographic-averaged *E*_*d*_ is used in the Eq. (1). The average threshold energy *E*_*d*_ for neutron-induced damage cross sections was selected to be 40 eV for Fe and 90 eV for W according to recommendations in Ref.^18^. Because the NRT-dpa model neglects ballistic in-cascade recombination effects, the experimental defect concentration can be lower than the calculated NRT-dpa value^19,20^. The recently proposed athermal recombination-corrected (arc-dpa) model includes the defect generation efficiency, *ξ(T),* and the third line of Eq. (1) is modified into the following form ^21^

2 $$N_{d} = 0.{8}T\xi (T)/\left( {{2}E_{d} } \right).$$

In the work^14^, it was shown that experimental data for neutron- and proton-irradiated Fe are better described by the arc-dpa model than the NRT-dpa model. Whereas experimental data for neutron- and proton-irradiated W are between NRT-dpa and arc-dpa predictions. In the present paper, we compare the arc-dpa and NRT-dpa predictions with experimental data of the primary radiation defects in W and Fe after irradiation with fission and energetic neutrons. This work highlights the potential of PALS technique for studying primary radiation damage, as well as for validation radiation damage models.

## Experimental

Cold-rolled W foils with purity of 99.97% produced by Plansee and ARMCO type Fe foils with purity of 99.8% produced by Goodfellow were mechanically polished to mirror-like state. Then W foils were recrystallized at 2000 K for 40 min in ultra-high vacuum with a base pressure lower than 2 × 10^–9^ mBar. The Fe foils were annealed at 1173 K for one hour in vacuum with a base pressure of 1 × 10^–6^ mBar. In the case of Fe, such heat treatment allows us to minimize the density of defects but conserves the α-phase of bcc Fe. The size of samples was 10 × 10 mm^2^ and their thickness was 100 and 200 μm for W and Fe, respectively.

Wide-spectrum high energy neutron irradiation was carried out at a source of fast neutrons with continuous neutron spectra up to 33 MeV generated by a p(35 MeV) beam incident on a thick Be target at the U-120 M cyclotron of Nuclear Physics Institute (NPI CAS) in Řež (see Ref.^22^ for details). The thickness of the Be target was 8 mm, which means that the protons were stopped inside the target. The W and Fe foils were irradiated in air in the position of 15 mm from the front of the Be target. The average current of the proton beam was 10 µA, which resulted in a neutron flux of about 10^15^ m^−2^ s^−1^ at the current sample location. The irradiation was carried out at room temperature. More information about the irradiation procedure is given in Ref.^14^.

Irradiation with fission neutrons was performed in the channels of H9/3 of the LVR-15 research reactor of the Research Centre Řež^23^. The neutron spectrum in the irradiation position was calculated by the neutron transport code MCNP6.1^24^ using the current fuel rods distribution. The neutron flux was estimated to be ~ 2.9 × 10^17^ m^−2^ s^−1^. The fraction of fast neutrons with energies of E > 0.1 MeV was calculated as 7.9% of the total neutron flux, meaning that the flux of fast neutrons in the fission neutron spectra was 2.3 × 10^16^ m^−2^ s^−1^. This flux is about 20 times higher than the NPI CAS neutron flux from the p(35 MeV)-Be source.

**Figure 1:**
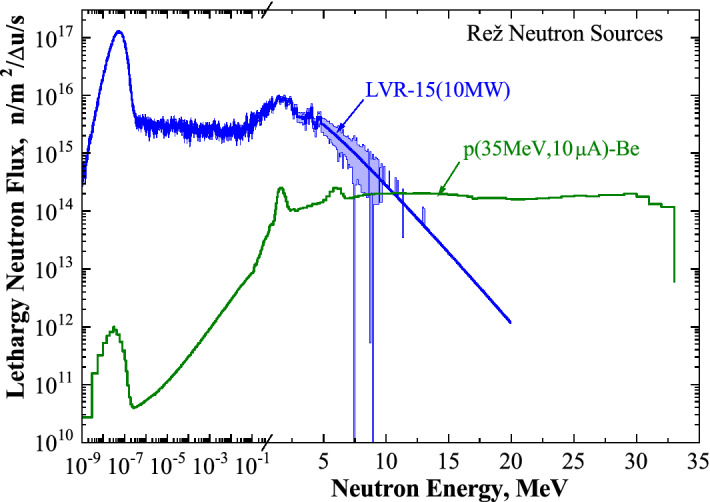
The neutron spectra generated in the channel H9/3 of the LVR-15 reactor and in the distance of 15 mm from the thick Be target bombarded with 35 MeV protons from the U-120 cyclotron.

The specimens were irradiated to a maximum fast neutron fluence of 1.56 × 10^21^ n/m^2^ for W and 5 × 10^20^ n/m^2^ for Fe, which corresponds to the displacement damage levels of 3.7 × 10^–5^ and 3.6 × 10^–5^ NRT-dpa for W and Fe, respectively. The temperature of the samples under irradiation was about 363–373 K. A comparison of the neutron spectra in the LVR-15 reactor and p(35 MeV)-Be neutron spectra is shown in Fig. 1.

The energy spectrum of neutrons in the reactor and neutrons produced by the p(35 MeV)-Be source are different. There is a significant fraction of thermal and epithermal neutrons in the reactor neutron spectrum. Thermal and epithermal neutrons do not have sufficient energy to produce a considerable concentration of vacancy-type defects. However, the thermal neutrons may cause transmutation via neutron capture reactions, whereas fast neutrons cause most of displaced atoms. Although the fraction of fast neutrons (E > 0.1 meV) in the total flux is 7.9%, this part of the spectrum contributes up to 90% of the total production of displacement damage.

**Figure 2:**
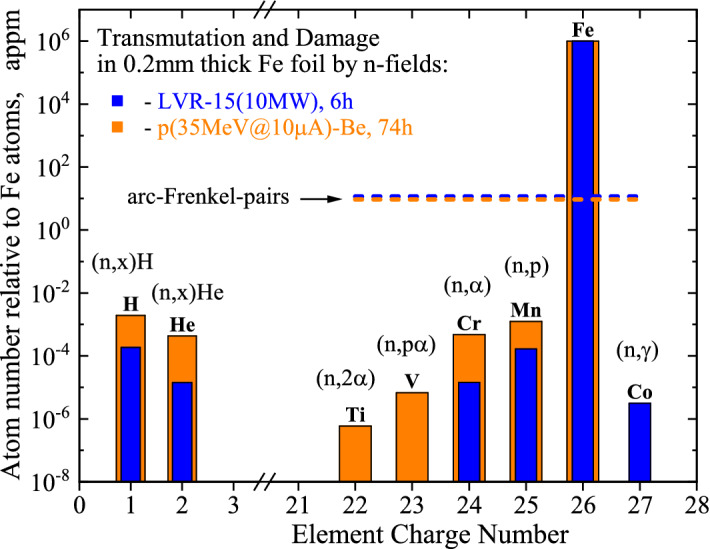
Calculated numbers of transmutants and displaced atoms (*arc*-Frenkel pairs) produced in W during irradiation in the LVR-15 reactor and p-Be source due to nuclear reactions.

**Figure 3:**
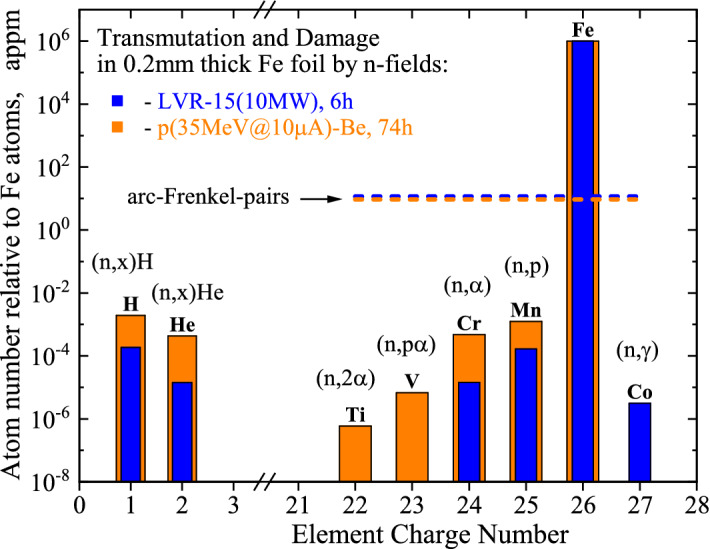
Calculated numbers of transmutants and displaced atoms (*arc*-Frenkel pairs) produced in Fe during irradiation in the LVR-15 reactor and p-Be source due to nuclear reactions.

We have estimated the nuclear transmutation of W and Fe into another elements for both neutron sources used in this work. For this purpose, the code FISPACT-II and JEFF-3.3 neutron data library^25^ were used. The number of transmutants accumulated during the maximum irradiation time (6 h in the LVR-15 reactor and 74 h in the p(35 MeV)-Be source) relative to the W and Fe atoms is shown in Figs. 2 and 3, respectively. For both materials, the softer reactor neutron spectrum results in a higher concentration of high Z-elements by the (n,γ) reactions than the accelerator driven neutron source. In contrast, the neutrons from the p(35 MeV)-Be source produce more low-Z elements and hydrogen or helium gases than the neutrons from the LVR-15 reactor.

The total relative inventory of the solid build-up elements generated in W and Fe in the LVR-15 reactor was calculated to be 4.1 × 10^–1^ appm and 1.8 × 10^–4^ appm, respectively. Irradiation of targets with the p(35 MeV)-Be neutrons produces 4.3 × 10^–4^ appm of new solid elements in W and 1.7 × 10^–3^ appm in Fe. The ratio of the concentration of solid build-up generated in W and Fe irradiated by reactor neutrons, trans^reactor^, to the concentration formed by neutrons from the p(35 MeV)-Be source, trans^p-Be^, is equal to

3 $${\text{trans}}^{{{\text{reactor}}}} /{\text{ trans}}^{{{\text{p}} - {\text{Be}}}} = {4}.{1} \times {1}0^{{ - {1}}} /{4}.{3} \times {1}0^{{ - {4}}} = { 1}0^{{3}}$$

in the case of W and

4 $${\text{trans}}^{{{\text{reactor}}}} /{\text{ trans}}^{{{\text{p}} - {\text{Be}}}} = {1}.{8} \times {1}0^{{ - {4}}} /{1}.{7} \times {1}0^{{ - {3}}} = { 1}0^{{ - {1}}}$$

in the case of Fe. The total concentration of solid build-up elements generated in W irradiated by reactor neutrons is significantly higher (about three orders of magnitude) than that in W irradiated by neutrons from the p(35 MeV)-Be source. In contrast, the concentration of solid transmutants in Fe due to the effects of reactor neutrons is lower compared to neutrons from the p(35 MeV)-Be source.

The calculated fractions of primary defects in W and Fe, i.e. Frenkel pairs of vacancies and interstitials survived in the PKA cascades are also plotted in Figs. 2 and 3. One can see that the total relative inventory of new build-up elements generated in W and Fe in the LVR-15 reactor and by wide-spectrum high energy neutrons from the p(35 MeV)-Be source is lower than the displacement damage predicted by the arc-dpa model. The ratio of the solid transmutants to the arc-Frenkel pairs reaches a maximum value of 17% in the case of W irradiation in the LVR-15 reactor due to the extremely high rate of rhenium production by thermal neutrons. Consequently, new elements produced by nuclear reactions during irradiation will have secondary impact on the radiation-induced defect evolution under current experimental conditions, such as decoration of defects or precipitation.

As it was mentioned above, the reactor flux of fast neutrons is about 20 times higher than the high-energy neutron flux from the p(35 MeV)-Be source. But there is a large fraction of high-energy neutrons in the range of 15–33 MeV in the NPI CAS neutron spectra from the p(35 MeV)-Be source. This fraction of high-energy neutrons is negligible in the fission neutron spectra (Fig. 1).

## Results

The intrinsic and radiation-induced defects in W and Fe were studied by PALS. A digital spectrometer^26^ with time resolution of 145 ps (FWHM ^22^Na) was employed for positron lifetime (LT) investigations. Positron source was made by deposition of 2 μl of ^22^NaCl water solution (iThemba Labs) with activity of ≈1.5 MBq on a 2 μm thick Mylar foil^27^**.** The diameter of the positron source spot was ≈1.5 mm and it was always positioned in the center of each sample measured. The details of PALS measurements can be found in Ref.^14^.

Results of decomposition of LT spectra are listed in Tables 1 and 2 for W and Fe samples, respectively.

**Table 1:** Positron lifetimes τ_i_ and relative intensities I_i_ of components measured by PALS in reference W samples and W samples irradiated with fission neutrons in the LVR-15 reactor at sample temperature of about 370 K.

Sample	Description	τ_1_ (ps)	I_1_ (%)	τ_2_(ps)	I_2_ (%)	C_cl_ (appm)	N_v_	C_v_ (appm)
WR	Recrystallized at 2000 K for 40 min	105(1)	100	–	–	–	–	–
WR_LVR1	n^0^-irradiated, F = 1.66 × 10^20^ m^−2^	95(5)	86(1)	244(3)	14(1)	1.4(2)	3.1(2)	4.5(5)
WR_LVR2	n^0^-irradiated, F = 2.1 × 10^20^ m^−2^	92(4)	84(1)	289(3)	16(1)	1.3(1)	5.0(2)	6.6(5)
WR_ LVR3	n^0^-irradiated, F = 5.1 × 10^20^ m^−2^	86(4)	74(1)	261(3)	26(1)	2.8(2)	3.8(2)	10.5(5)
WR_ LVR4	n^0^-irradiated, F = 1.56 × 10^21^ m^−2^	70(5)	54(2)	245(5)	46(2)	7.0(5)	3.4(5)	24(1)

The fluence of fast neutrons (E > 0.1 MeV), F, is also shown. The experimental errors expressed in units of the last significant digit are given in parentheses. The physical parameters calculated from PALS data:

*C*_*cl*_ concentration of vacancy clusters, *N*_*v*_ average number of vacancies in a cluster, *C*_*v*_* = C*_*cl*_* N*_*v*_ total concentration of vacancies.

**Table 2:** Positron lifetimes τ_i_ and relative intensities I_i_ of components measured by PALS in reference Fe samples and Fe samples irradiated with fission neutrons in the LVR-15 reactor at sample temperature of about 370 K.

Sample	Description	τ_1_ (ps)	I_1_ (%)	τ_2_(ps)	I_2_ (%)	C_cl_ (appm)	N_v_	C_v_ (appm)
Fe	Annealed at 1173 K for 1 h	107(5)	100	–	–	–	–	–
Fe_LVR1	n^0^-irradiated, F = 1.66 × 10^20^ m^-2^	102(3)	90.4(5)	190(5)	9.6(5)	1.4(2)	1.4(2)	2.0(4)
Fe_LVR2	n^0^-irradiated, F = 2.1 × 10^20^ m^-2^	98(2)	80(2)	195(4)	20(2)	2.7(1)	1.7(2)	4.6(5)
Fe_LVR3	n^0^-irradiated, F = 5.1 × 10^20^ m^-2^	88(2)	68(1)	197(1)	32(1)	5.1(1)	1.8(2)	9.2(5)

The fluence of fast neutrons (E > 0.1 MeV), F, is also shown. The physical parameters calculated from PALS data are listed in the table as well. The experimental errors expressed in units of the last significant digit are given in parentheses. Notation is the same as in Table 1.

Annealed non-irradiated W and Fe are characterized by single component PALS spectrum. The lifetime τ_1_ of this component is (105 ± 1) and (107 ± 5) ps, for W and Fe, respectively (see Tables 1 and 2). These values agree well with the bulk positron lifetime for W ^28^ and Fe^29^. Hence, the component with lifetime τ_1_ represents a contribution of free positrons de-localized in the lattice. It means that the defect concentration in the annealed W and Fe is lower than 1 appm since virtually all positrons annihilate from the de-localized state in the lattice^30–33^.

The PALS spectra of both W and Fe samples irradiated in the LVR-15 reactor with fission neutron spectrum to a fluence of 2.12 × 10^21^ n/m^2^ (1.67 × 10^20^ n/m^2^ of fast neutrons with an energy of E > 0.1 MeV) contain a new component with longer lifetime τ_2_ = (190 ± 5) ps for Fe and τ = (244 ± 3) ps for W (see Tables 1, 2). This component can be attributed to positrons trapped at radiation-induced defects. Comparing experimental lifetimes with results of ab-initio theoretical calculations^32–35^**,** one can conclude that small vacancy clusters consisting on average of 1–2 and 4 vacancies were created by fission neutron irradiation in Fe and W, respectively. Dislocation-type defects were not detected neither in W nor in Fe up to the fluence of 5 × 10^20^ n/m^2^.

In general, the transmutation elements, resulting from the interaction of low-energy neutrons (present in the spectra of fission neutrons) with a solid, can decorate the radiation-induced defects. With a high ratio of thermal neutrons to fast neutrons, interstitial impurities as a result of transmutation can reduce the lifetime of positrons trapped in vacancy-type defects by decorating these defects^36^. Hence, if the concentration of transmutation products is comparable to dpa, the size of radiation-induced defects might be larger than the size obtained by comparing the experimental data with ab-initio theoretical calculations. However, for low fluences used in this study the effects of transmutation impurities are negligible since the concentration of transmutation products is substantially lower than dpa. Consequently, both fission and energetic neutron irradiations of W and Fe samples introduced predominantly small vacancy clusters. The driving force for the agglomeration of individual vacancies into vacancy clusters is a reduction of the surface energy. Agglomeration of radiation-induced vacancies is facilitated by the fact that neutron irradiation produces vacancies in cascades, i.e. located close to each other. Agglomeration of such vacancies into clusters is relatively easy, since it does not require diffusion to a long distance^14^.

**Figure 4:**
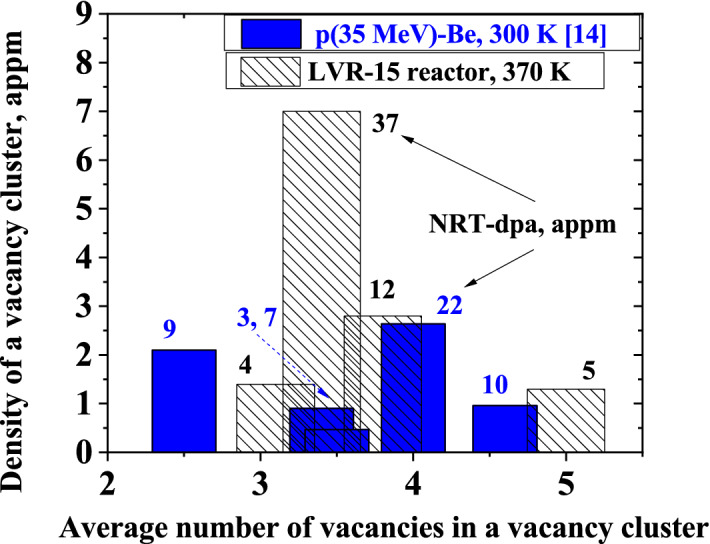
Experimental data of the mean size of radiation-induced vacancy clusters in W and their density after irradiation in the LVR-15 reactor in comparison with high-energy p(35 MeV)-Be neutron irradiation^14^ for each irradiation fluence indicated for the NRT-dpa values in appm.

The mean size of radiation-induced vacancy clusters in W after fission and high-energy p(35 MeV)-Be neutron irradiations is compared in Fig. 4. The NRT-dpa is indicated by labels in the figure. The temperature of samples was ~ 300 K and ~ 370 K during irradiation with neutrons from the p(35 MeV)-Be source and in the LVR-15 reactor, respectively. Thermally activated migration of vacancies in W is negligible at these temperatures^2,37^. Unlike low-Z materials, for example Fe, which require irradiation and subsequent measurement at cryogenic temperatures in order to neglect the impact of thermal effects on the defect recovery^30^, W has the advantage of high melting temperature and, therefore, migration of vacancy-type defects is insignificant even at 400 K. Since migration of interstitials occurs at significantly lower temperatures compared to vacancies, some vacancy-type defects in W were likely removed by recombination with migrating interstitials which are mobile well below room temperature.

From Fig. 4, one can conclude that both fission neutrons from the LVR-15 reactor and high-energy neutrons from the p(35 MeV)-Be source create in W vacancy clusters containing on average 3–5 vacancies.

**Figure 5:**
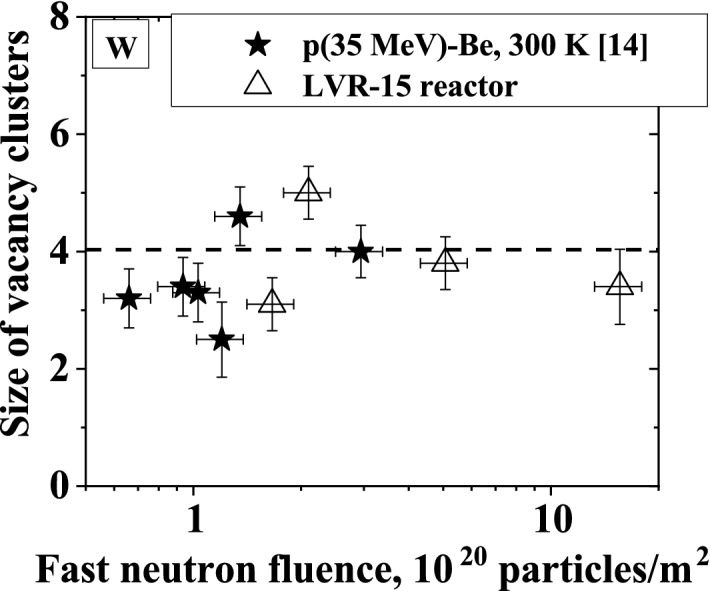
Experimental data of the mean size of vacancy clusters in W induced by irradiation with neutrons from p(35 MeV)-Be source^14^ and neutrons in LVR-15 reactor as a function of the irradiation fluence. Dashed line is weighted average of the mean size of vacancy clusters.

Figure 5 shows experimental data of the average size of vacancy clusters in W induced by irradiation with neutrons from p(35 MeV)-Be source and neutrons in the LVR-15 reactor as a function of irradiation fluence. The average size of vacancy clusters for both types of irradiation is comparable up to the fluence of 10^21^ n/m^2^. This is because vacancies are immobile at the irradiation temperature 300–370 K and the fluences are too low for cascade overlap events. According to SRIM calculations, there are no cascade overlap events at such fluences^14^. Moreover, the average size of vacancy clusters does not remarkably change with increasing fluence.

**Figure 6:**
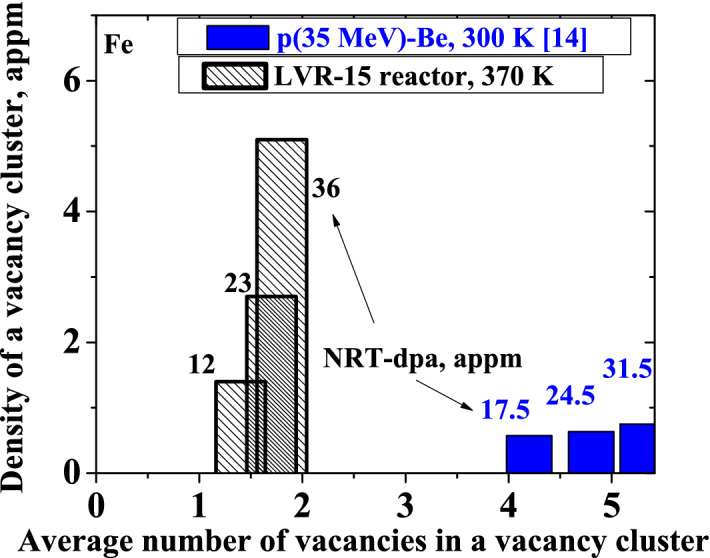
Experimental data of the mean size of radiation-induced vacancy clusters in Fe and their density after irradiation in the LVR-15 reactor in comparison with high-energy p(35 MeV)-Be neutron irradiation^14^ for each irradiation fluence indicated in NRT-dpa values in appm.

Vacancy-related defects in the Fe samples irradiated in the nuclear reactor are remarkably smaller than clusters created by p(35 MeV)-Be neutrons (Fig. 6). The density of radiation-induced defects increases with increasing fluence/dpa. For Fe irradiated with fission neutrons, the mean size of vacancy clusters increases from 1–2 vacancies to 2 vacancies per cluster with NRT-dpa increasing from 12 × 10^–6^ to 36 × 10^–6^. For Fe irradiated with high-energy neutrons from the p(35 MeV)-Be source, the mean size of vacancy clusters grows from 4 up to 5–6 vacancies per cluster with NRT-dpa increasing from 17 × 10^–6^ to 31 × 10^–6^.

## Discussion

### Cluster size after fission and high-energy neutron irradiation

As follows from PALS measurements, the irradiation of W with neutrons from both the p(35 MeV)-Be source and fission reactor creates in W small vacancy clusters of approximately the same size. Irradiation of Fe in the LVR-15 reactor creates vacancy clusters with (i) smaller size and (ii) higher concentration compared to irradiation with neutrons from the p(35 MeV)-Be source at the same dpa. In both irradiation spectra, the vacancy clusters in Fe appear to increase in the size and the density with increasing fluence. The increase in the size is evidence for vacancy flow to vacancy clusters produced in earlier stages of the irradiation.

Note, that according to our SRIM simulations, the mean distance between displacements is in the order of 0.1 μm even for the highest fluence used in the present work. Hence, cascades do not overlap with each other. Since vacancies are immobile in W at the irradiation temperatures and the maximum dose of ~ 3 × 10^–5^ dpa precludes cascade overlap effects, flux (or dose rate) should not cause vacancy growth. Furthermore, flux would not have any effect on in-cascade local vacancy concentration. The present data also showed that the vacancy cluster size in W is independent of the fluence for conditions where cascade overlap can be neglected and thermally activated migration of vacancies does not occur. A similar size of vacancy clusters in W has been measured for all fluences studied.

The smaller size of radiation-induced defects in Fe with higher density after fission neutron irradiation compared to p(35 MeV)-Be neutrons can be attributed to (i) a high fraction of high-energy neutrons in the neutron spectra from the p(35 MeV)-Be source and (ii) flux effect caused by thermally activated migration of radiation-induced vacancies. High energy primary knock-on atoms (PKAs) transfer substantial kinetic energy to the host atoms, leading to displacement cascades that can be significantly greater than the defect migration enthalpies. As a consequence, both pronounced defect agglomeration and recombination during the cascade quench phase take place. In the case of Fe irradiated at room temperature, thermal effects play noticeable role in radiation-induced defect recombination and agglomeration. Migration of mono-vacancies was observed already at 220 K in electron-irradiated Fe (annealing stage III) and at 180 K in Fe irradiated with neutrons^30^**.** In our current experiments, the samples were irradiated in the temperature range of 300–373 K, which is far above the stage III. Therefore, vacancies are mobile at the temperature of irradiation. Radiation-induced vacancies that do not recombine with interstitials either disappear by diffusion to sinks at the grain boundaries and on the surface or can agglomerate into clusters that are immobile at the irradiation temperature. A migrating vacancy can be trapped in an already formed cluster increasing thereby its size. Consequently, contrary to W, in Fe samples the size of vacancy clusters increases with increasing fluence. Low flux leads to fewer number of radiation-induced vacancies created in the sample per unit time. This means that the probability that a vacancy disappears by diffusion into sinks or recombines with the interstitial is increased. This results in the formation of a smaller number of vacancy clusters with larger average size compared to high flux. As the flux increases, the concentration of point defects introduced per unit time increases as well. Because their thermal mobility remains unchanged, the probability that vacancies meet each other and form immobile cluster increases. Since these clusters are immobile at the irradiation temperature, they cannot merge with each other. As a consequence, a higher density of vacancy clusters with a smaller size is formed.

According to molecular dynamic (MD) calculations, the vacancy cluster size distribution in Fe shifts to larger size as the cascade energy increases from 10 to 50 keV^38^. The cascade energy of 10 and 50 keV corresponds to neutron energy of about 0.4 and 1 MeV, respectively. The conclusion of the MD calculations is supported by clearly separated clusters of vacancies by size for different cascade energies in the case of Fe, namely single vacancies and divacancies formed after irradiation with fission neutrons and vacancy clusters with 4–5 vacancies introduced by irradiation with a wide-spectrum of high-energy neutrons, see Fig. 6.

In the case of W, no clear effect of the cascade energy on the average vacancy size was reported in the MD calculations when the cascade energy increases from 1 to 30 keV^39^**.** However, the vacancy cluster fraction slightly increases as the cascade energy increases from 30 to 100 keV. The cascade energy of 30 keV and 100 keV corresponds to neutron energy of about 1 and 4 MeV, respectively. We did not observe any noticeable difference in the average vacancy size between reactor neutrons and a wide-spectrum high-energy neutrons irradiations of W up to 37 appm NRT-dpa, see Figs. 4 and 5.

### Comparison with theory

The NJOY-2016^40^ was employed for calculation of the NRT- and arc-dpa cross sections for neutron irradiation. The code processes the evaluated cross section data and calculates the damage energy available for atom displacement from lattice site using the embedded NRT model. In order to calculate radiation damage within the arc-dpa approach, the primary defect survival function was incorporated into the NJOY-2016 code^41^. The primary defect survival function was constructed using available results of MD simulations^21^. Binary collision approximation (BCA) was used for the nuclear reaction recoil energies above 150–200 keV, where the results of MD simulations are not yet available^42^. Accurate cross-sections of the neutron reactions are crucial for the NRT- and arc- dpa calculations. The latest versions of the major evaluations of neutron cross-sections, namely, US Evaluated Nuclear Data File ENDF-VIII.0^43^ and Joint Evaluated Fission and Fusion library JEFF-3.3^44^, were used in the present work. Table 3 shows the neutron-induced NRT-dpa and arc-dpa cross-sections for W and Fe calculated from the full nuclear reaction data.

**Table 3:** Measured and calculated neutron-induced dpa cross-sections for W and Fe averaged in the neutron spectrum produced in the LVR-15 reactor and the p(35 MeV)—Be source.

Type of spectrum: effective energy	Experimental dpa, barn	Calculated arc-dpa (NRT-dpa), barn		Experim./Calcul arc-dpa (NRT-dpa)
		ENDF/B-VIII.0	JEFF-3.3	
**Tungsten**				
U-235(n,f): 2.4 ± 1.3 MeV	121 ± 27 Guinan’82 (T < 10 K)^13^ 156 ± 62 Klabunde'82 (T < 10 K)^12^	51 (184)	58 (221)	2.4 ± 0.4 (0.63)^a^
LVR-15 reactor, 1.9 ± 1.0 MeV	205 ± 30 this work, (T ≈ 370 K)	50 (160)	53 (184)	3.9 ± 0.6 (1.11)^a^
p(35 MeV)—Be: 15.7 ± 10.0 MeV	330 ± 15^14^ (T ≈ 300 K)	97 (371)	167 (659)	2.0 ± 0.1 (0.50)^a^
D–T (RTNS-II): 14.9 ± 0.3 MeV	428 ± 97 Guinan’82 (T < 10 K)^13^	112 (438)	211 (877)	2.0 ± 0.2 (0.49)^a^
**Iron**				
U-235(n,f): 2.4 ± 1.3 MeV	224 ± 23 Horak’73 (T < 10 K)^45^ 264 ± 44 Takamura’85 (T < 10 K)^46^ 260 ± 43 Wallner’88 (T < 10 K)^47^	262 (856)	253 (826)	1.00 ± 0.2 (0.33)^b^
LVR-15 reactor, 2.2 ± 1.3 MeV	115 ± 20 this work, (T ≈ 370 K)	237 (729)	234 (714)	0.49 ± 0.08 (0.16)^b^
p(35 MeV)—Be: 11.1 ± 9.5 MeV	220 ± 10^14^ (T ≈ 300 K)	546 (1749)	499 (1593)	0.44 ± 0.10 (0.14)^b^
D–T (RTNS-II): 14.9 ± 0.3 MeV	–	797 (2543)	709 (2245)	–

The calculations were performed using arc-dpa and NRT-dpa models with nuclear data from ENDF/B-VIII.0 and JEFF-3.3. The known previous measurements at cryogenic temperatures in fission reactors and with the D-T source are given for comparison.

Comments: ^a^ratio to JEFFF-3.3; ^b^ratio to ENDF/B-VIII.0.

In Table 3 the theoretical cross-sections are compared with experimental values determined from PALS measurements by dividing the total concentration of vacancies, *c*_*v*_, by the irradiation fluence. From inspection of the Table 3, one can conclude that the arc-dpa equals ≈ 1/3 of the NRT-dpa in the energy range of practical interest. In order to compare the calculated quantities with the neutron-induced cross-sections determined experimentally in the present work as well as in the available literature using fission reactors^12,13,45–47^ and D–T generator^13^, the theoretical NRT- and arc-dpa cross-sections were folded with the neutron spectra of the corresponding facilities. It has to be mentioned that in contrast to Fe, the cross section calculated for W using nuclear data libraries ENDF/B-VIII.0 and JEFF-3.3 differs roughly in two times.

**Figure 7:**
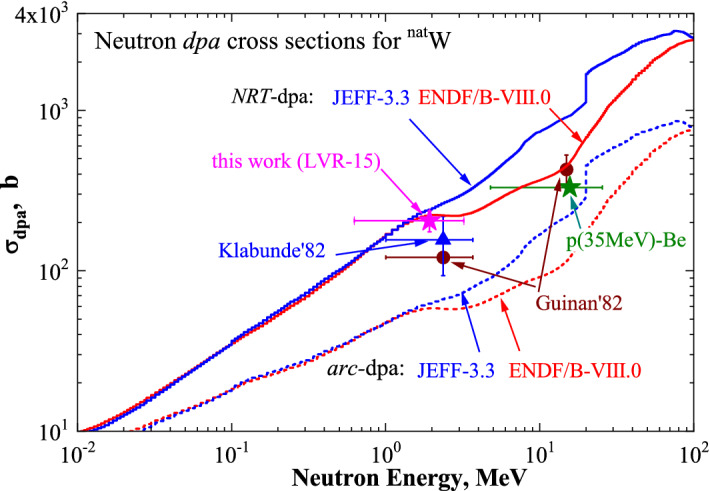
Experimental neutron-induced cross-sections for W measured in the present work after irradiation in the LVR-15 reactor at T ≈ 370 K and neutrons from the p(35 MeV)-Be source at T ≈ 300 K reported in Ref.^14^, as well as those that are known from the literature at T < 10 K, namely, Kladunde’82^12^ and Guinan’82^13^ (symbols with error bars). Calculated neutron-induced NRT-dpa (solid lines) and arc-dpa (dashed lines) cross sections for W using neutron reaction data from ENDF/B-VIII.0 and JEFF-3.3 are also shown for comparison.

**Figure 8:**
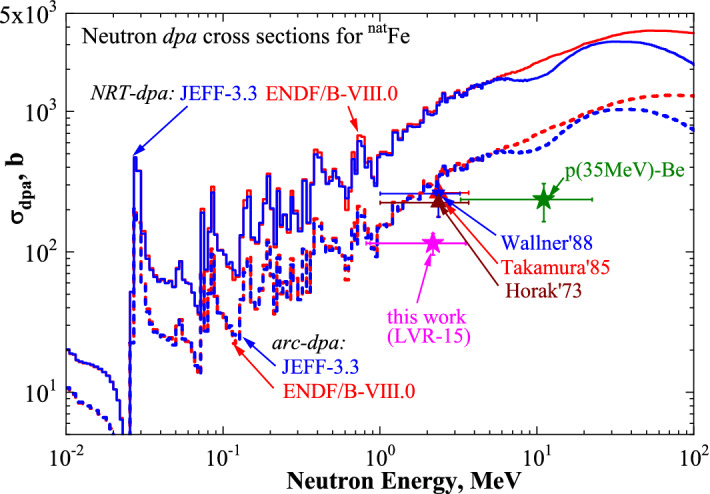
Experimental neutron-induced cross-sections for Fe measured in the present work after irradiation in the LVR-15 reactor at T ≈ 370 K and neutrons from the p(35 MeV)-Be source at T ≈ 300 K reported in Ref.^14^, as well as those that are known from the literature at T < 10 K, namely, Horak’73^45^**,** Takamura’85^46^**,** and Wallner’88^47^ (symbols with error bars). Calculated neutron-induced NRT-dpa (solid lines) and arc-dpa (dashed lines) cross sections for Fe using neutron reaction data from ENDF/B-VIII.0 and JEFF-3.3 are also shown for comparison.

The calculated and experimental NRT- and arc-dpa neutron irradiation cross-sections are compared in Figs. 7 and 8 for W and Fe, respectively.

For W irradiated with fission neutrons, the experimental data are better described by the NRT-dpa model than the arc-dpa model. The measured dpa cross-section of high-energy neutrons from the p(35 MeV)-Be source in the case of W^14^ is in a reasonable agreement with either the NRT-dpa model using neutron reaction data from ENDF/B-VIII.0 library, or with the arc-dpa prediction using neutron reaction data from JEFF-3.3 library (Fig. 7). Considering also the experimental data from the available literature^12,13^, one can conclude that the experimental neutron-induced cross-sections for W are straddling between the two models confirming the conclusion reported in Ref.^14^. Measured values for defect production efficiency in W may be underestimated because of self-interstitial defects, which are created as well as vacancies and are mobile already below room temperature. At low irradiation temperatures and low initial sink density, the recombination induced by the diffusion of interstitials is responsible for the density of vacancy-type of defects. Migrating interstitials may recombine with immobile vacancies or be trapped in a vacancy cluster, thus, reducing its size. Increasing the fluence, interstitials first find the sinks, which leads to a decrease in their concentration and a corresponding increase in the net vacancy concentration as observed experimentally. Therefore, because of migrating self-interstitial defects the discrepancy between measured and calculated dpa for W may be greater than indicated in Table 3. The general trend of radiation-induced defect recovery agrees well for both neutron and electron irradiations, especially with regard to a significant recovery stage below 25 K. The recovery of the electrical resistivity of W following low temperature electron irradiation was reported to be ~ 50–70% defect recovery after annealing through Stage I (up to 100 K)^48,49^. About 50% of initial radiation damage in thermal neutron-irradiated W after cryogenic irradiation was recovered by 300 K as reported in Ref.^50^. In the current study, no big difference between radiation-induced defect density in W irradiated at 300 K (determined by positron annihilation spectroscopy techniques) and prior defect production studies at 10 K (used electrical resistivity techniques) is observed. This can be due to various methods of measurements, various purity of W, irradiation conditions (damage rate versus annealing time), transmutation effects, etc. One possible reason may be that dissolved impurity atoms presented in our samples could suppress the radiation-induced defect recovery^51^.

Figure 8 shows the neutron-induced cross-sections determined experimentally in the present work for Fe irradiated with fission neutrons in the LVR-15 reactor at T ≈ 370 K. The data known from the literature are plotted in the figure as well. The experimental data of fission neutron irradiation of Fe at T < 10 K^45–47^ are in a good agreement with the calculated spectrum averaged arc-dpa cross sections. In our previous publication^14^**,** the fast reactor spectrum averaged arc-dpa for Fe, measured by Horak and Blewitt^45^**,** was derived from the overview table published by P. Jung^52^. It resulted in the value of 136 barn, which is lower by a factor of two than other reactor measurement^46,47^. In contrast to this, the dpa value measured by the electrical resistivity change in Ref.^45^ is 224 barn. Now, we use this value since it corresponds to the original author’ data and well agrees with other reactor measurements^46,47^**.**

The arc-dpa model is in a good agreement with most of the experimental data at low irradiation temperatures of T < 10 K. It indicates that annealing of the hot recoils in cascade of collisions in Fe is the dominating factor of the recovery of primary radiation-induced defects. This proves the validity of the arc-dpa cross section calculated using the primary defect surviving function based on the results of the state-of-art MD simulations.

The cross-section of fission neutrons in the LVR-15 reactor measured in this work for Fe is lower than the arc-dpa prediction, similar to the data for neutrons from the p(35 MeV)-Be source. This is due to the thermal annealing of defects. Thermally activated recovery of defects in Fe that occurs already at ambient temperature reduces the radiation damage predicted by the arc-dpa model by a factor of about two. The concentration of radiation-induced defects is significantly reduced due to recombination effects occurring at the subsequent stages of annealing: I (below 0.1*T*_*m*_)—recombination of close Frenkel pairs associated with self-interstitial atom motion, II—recombination of remote Frenkel pairs (*T *≈ 0.15*T*_*m*_), III—migration of mono-vacancies into sinks and their agglomeration into clusters (*T *≈ 0.15–0.2*T*_*m*_), where *T*_*m*_ is the melting temperature^2,30,37,53–56^. Therefore, not only athermal vacancy-interstitial recombination considered in the arc-dpa model, but also thermally activated diffusion of point defects to sinks contributes to a significant recovery of radiation-induced vacancies and interstitials^2,20,30,45–47^.

## Conclusions

We initiated a study of the effect of different neutron spectra on the cluster size of primary radiation-induced defects in bcc Fe and W. PALS technique has been applied to study the size and concentration of vacancy-type radiation-induced defects and to explore the validity of the damage models for fission neutron spectra and a wide range high energy neutron spectra.

With irradiation up to the fluence of about 1 × 10^21^ n/m^2^, dislocation-type defects produced by irradiation were not detected neither in W nor in Fe, only vacancy-type defects were detected. It was found that irradiation of W with neutrons produced in a nuclear reactor and with high-energy neutrons from a p(35 MeV)-Be generator leads to the formation of radiation-induced vacancy clusters with comparable mean size of ≈ 4 vacancies.

Fe samples irradiated with neutrons from the p(35 MeV)-Be source contained vacancy clusters with a larger size and lower density compared to the samples irradiated with fission neutrons. One reason for this is higher flux in the case of fission neutron irradiation that leads to a higher density of vacancy clusters with a smaller size. The second reason for the larger clusters of vacancies with lower concentrations formed during high-energy neutron irradiation is the higher energy of PKAs in the neutron spectra of the p(35 MeV)-Be source compared to fission neutrons. High energy PKAs transfer substantial kinetic energy to the host atoms, resulting in an energetic displacement cascade. This enhances both defect agglomeration and recombination during the cascade quenching phase. As a consequence, the actual damage of samples may be less than expected from the NRT-dpa model. This is consistent with the concept of the athermal recombination corrected (arc-dpa) model. Moreover, thermally activated diffusion of defects even at 300 K leads to defect recombination, which significantly (by a factor of about two) reduces the concentration of radiation-induced defects predicted by the arc-dpa model. Thus, different neutron spectra and different fluxes can lead to different cluster sizes of radiation-induced defects, despite the same dpa. The sample temperature has a stronger effect on radiation-induced defect size than the irradiation fluence. Different cluster sizes, which are not taken into account in the dpa models, can invariably contribute to changes in the microstructure and, consequently, the physical properties of irradiated materials. This means that the physics of primary radiation effects in materials is still not fully known and requires further study through a combination of modeling and experimental efforts. The present data serve as a basis for developing an improved concept of the displacement process.

## Methods

1. *Sample preparation *Cold-rolled W foils with a thickness of 100 μm and purity of 99.97% produced by Plansee and ARMCO type Fe foils with a thickness of 200 μm and purity of 99.8% produced by Goodfellow were mechanically polished to mirror-like state. Then W foils were recrystallized at 2000 K for 40 min in ultra-high vacuum with a base pressure lower than 2 × 10^–9^ mBar. The Fe foils were annealed at 1173 K for 1 h in vacuum with a base pressure of 1 × 10^–6^ mBar. In the case of Fe, such heat treatment allows us to minimize the density of defects but persist α-phase of bcc Fe.
2. *Irradiation with wide-spectrum high energy neutrons* Wide-spectrum high energy neutron irradiation was carried out at the source of fast neutrons with continuous neutron spectra up to 35 MeV generated by a p(35 MeV) beam incident on a thick Be target at the U-120M cyclotron of Nuclear Physics Institute (NPI CAS) in Řež. The detailed description is given in Ref.^22^. The neutron flux was 10^15^ n/m^2^s. The neutron irradiation was carried out at room temperature. The neutron fluence on the foils was 0.4 × 10^20^, 1.0 × 10^20^, 1.4 × 10^20^, and 1.8 × 10^20^ n/m^2^.
3. *Irradiations with fission neutrons* Irradiation with fission neutrons has been performed in the channels of H9/3 of the LVR-15 research reactor of the Research Centre Řež. The detailed description is given in Ref.^23^. The neutron spectrum in the irradiation position was calculated by the neutron transport code MCNP6.1 using the current fuel rods distribution. The neutron flux was estimated to be ~ 2.9 × 10^17^ m^−2^ s^−1^. The fraction of fast neutrons with energies of E > 0.1 MeV was calculated as 7.9% of the total neutron flux, meaning that the flux of fast neutrons in the fission neutron spectra was 2.3 × 10^16^ m^−2^ s^−1^. The temperature of the samples under irradiation was about 363–373 K. The fluence of fast neutrons on the foils was 1.67 × 10^20^, 2.1 × 10^20^, 5.1 × 10^20^ n/m^2^, and 1.56 × 10^21^ n/m^2^.
4. *Defect characterization* Positron annihilation lifetime spectroscopy (PALS) measurements and a computer simulation of PALS spectra were used for intrinsic and radiation-induced defects investigation. A digital spectrometer with time resolution of 145 ps (FWHM ^22^Na) was employed for positron lifetime (LT) investigations. Positron source was made by deposition of 2 μl of ^22^NaCl water solution (iThemba Labs) with activity of ≈ 1.5 MBq on a 2 μm thick Mylar foil**.** The diameter of the positron source spot was ≈ 1.5 mm and it was always positioned in the center of each sample measured. At least 10^7^ positron annihilation events were accumulated in LT spectra which were decomposed using a maximum likelihood based procedure. The source contribution consisted of two weak components which come from positrons annihilated in the source spot and the covering Mylar foil and exhibit lifetimes of ~ 368 ps and ~ 1.5 ns. Corresponding intensities of the source contribution components were ~ 6% and ~ 1% for Fe and ~ 10% and ~ 1.7% for W, respectively. The details of these PALS measurements can be found in Refs.^26,27^.
5. *Nuclear transmutation calculations *The nuclear transmutation of W and Fe into another elements was calculated using the code FISPACT-II and JEFF-3.3 neutron data library.
6. The neutron induced NRT- and arc-dpa cross sections were calculated by the NJOY-2016 code using the evaluated neutron reaction data from the latest versions of the main evaluations ENDF-VIII.0 and JEFF-3.3. The lattice displacement energy E_d_ was selected to be 40 eV for Fe and 90 eV for W. The primary defects (Frenkel pairs) survival function which impact on arc-dpa was taken for Fe and W from the Primary Radiation Group of the Organisation for Economic Co-operation and Development (OECD)^21^ by fitting to the selected molecular dynamic (MD) simulations. Above the PKA energies of 150–200 keV, where the results of MD simulations are not presently available, the combination of the MD and binary collision approximation (BCA) calculations was used to compute defect survival efficiency. The displacement cross sections files are available on: https://www-nds.iaea.org/CRPdpa/, https://www.oecd-nea.org/dbdata/JEFF33/.

## Data Availability

The authors declare that all data supporting the findings of this study are available from the authors on request.
